# Antimicrobial, Probiotic, and Immunomodulatory Potential of *Cannabis sativa* Extract and Delivery Systems

**DOI:** 10.3390/antibiotics13040369

**Published:** 2024-04-17

**Authors:** Anna Stasiłowicz-Krzemień, Daria Szymanowska, Piotr Szulc, Judyta Cielecka-Piontek

**Affiliations:** 1 Department of Pharmacognosy and Biomaterials, Faculty of Pharmacy, Poznan University of Medical Sciences, Rokietnicka 3, 60-806 Poznan, Poland; daria.szymanowska@up.poznan.pl; 2 Department of Biotechnology and Food Microbiology, Poznan University of Life Sciences, 48 Wojska Polskiego Street, 60-627 Poznan, Poland; 3 Department of Agronomy, Poznań University of Life Sciences, Dojazd 11, 60-632 Poznan, Poland; piotr.szulc@up.poznan.pl; 4 Department of Pharmacology and Phytochemistry, Institute of Natural Fibres and Medicinal Plants, Wojska Polskiego 71b, 60-630 Poznan, Poland

**Keywords:** *Cannabis sativa*, cannabidiol, antibacterial, probiotic, immunomodulatory, immunostimulatory

## Abstract

The compounds present in hemp show multidirectional biological activity. It is related to the presence of secondary metabolites, mainly cannabinoids, terpenes, and flavonoids, and the synergy of their biological activity. The aim of this study was to assess the activity of the Henola *Cannabis sativae* extract and its combinations with selected carriers (polyvinyl caprolactam–polyvinyl acetate–polyethylene glycol graft copolymer, magnesium aluminometasilicate, and hydroxypropyl-β-cyclodextrin) in terms of antimicrobial, probiotic, and immunobiological effects. As a result of the conducted research, the antimicrobial activity of the extract was confirmed in relation to the following microorganisms: *Clostridium difficile*, *Listeria monocytogenes*, *Enterococcus faecalis*, *Staphylococcus aureus*, *Staphylococcus pyrogenes*, *Escherichia coli*, *Klebsiella pneumoniae*, *Salmonella typhimurium*, *Pseudomonas aereuginosa*, and *Candida albicans* (microorganism count was reduced from ~10^2^ CFU mL^−1^ to <10 CFU mL^−1^ in most cases). Additionally, for the system with hydroxypropyl-β-cyclodextrin, a significant probiotic potential against bacterial strains was established for strains *Lactobacillus acidophilus*, *Lactobacillus casei*, *Lactobacillus plantarum*, *Lactobacillus brevis*, *Lactobacillus rhamnosus*, *Lactobacillus reuteri*, *Pediococcus pentosaceus*, *Lactococcus lactis*, *Lactobacillus fermentum*, and *Streptococcus thermophilus* (microorganism count was increased from ~10^2^ to 10^4^–10^7^). In terms of immunomodulatory properties, it was determined that the tested extract and the systems caused changes in IL-6, IL-8, and TNF-α levels.

## 1. Introduction

*Cannabis sativa* L., a plant belonging to the *Cannabaceae* family, is used for various purposes, e.g., in the food industry, for medicinal and cosmetic applications, production of textiles and ropes, manufacturing biodegradable plastics, and paper production [1]. One of the intriguing aspects of this plant is its potential antimicrobial properties, which have gained attention in recent years due to the increasing concerns about antibiotic resistance and the need for new sources of antimicrobial agents. *Cannabis sativa* contains a complex of secondary plant metabolites, many of which contribute to its potential antimicrobial effects. The primary classes of compounds found in cannabis are cannabinoids, terpenes, and flavonoids [2]. Cannabinoids are unique chemical compounds found in cannabis that interact with the endocannabinoid system in humans and other animals. The most well-known cannabinoid is Δ^9^-tetrahydrocannabinol (THC), which has analgesic, anticonvulsant, antiepileptic, sleep-improving, appetite-stimulating, and antiemetic effects through partial agonism on CB_1_ and CB_2_ receptors and agonism on G protein-coupled receptor 55 (GPR55) and GPR18, while also displaying antinociceptive and antiemetic actions via antagonism on 5-hydroxytryptamine (5-HT3A) receptors [3,4]. The other most known cannabinoid is cannabidiol (CBD), which has various therapeutic effects, including anti-inflammatory, anxiolytic, and antiepileptic properties, without inducing psychotropic effects like THC [5]. CBD modulates CB_1_ and CB_2_ receptors, resulting in anxiolytic, antidepressant, vasorelaxant, antiseizure, anti-inflammatory, anticancer, and neuroprotective effects, while also influencing GPR55, fatty-acid amide hydrolase (FAAH), 5-HT1A, α-1, and α-2 adrenoceptors, peroxisome proliferator-activated receptors (PPAR-γ), glycine receptors (GlyR-α1 and GlyR-α3), γ-aminobutyric acid receptor (GABA-A), and transient receptor potential vanilloid (TRPV1 and TRPV2) for sleep induction, stress reduction, analgesia, antiemesis, and cardiovascular benefits [4,6,7,8]. Another cannabinoid with growing interest is cannabigerol, CBG, which interacts primarily with cannabinoid receptors CB_1_ and CB_2_, along with α-2 adrenoceptors and 5-HT1A receptors, influencing neurotransmitter release and immune function. It has anti-inflammatory, antioxidant, antibacterial, and neuroprotective effects [9,10]. Cannabinoids might be used in neuropathic pain, spasticity in multiple sclerosis, nausea, vomiting, cancer pain, and certain behavioral symptoms in dementia [11,12,13,14,15,16,17]. Additionally, cannabis use might be beneficial in patients with inflammatory bowel diseases, anxiety disorders, and sleep disorders [18,19,20,21]. The essential oil of cannabis comprises mono- and sesquiterpenes, encompassing various chemical groups, including alcohols, aldehydes, and ketones, as well as acyclic, monocyclic, and bicyclic structures [2,22]. Cannabis terpenes such as myrcene, pinene, limonene, and caryophyllene, alongside terpinolene, humulene, and linalool, contribute to the diverse aromatic profile of cannabis strains, ranging from woody and spicy to floral and herbal scents [23,24]. Terpenes can interact with receptors and enzymes in the body, potentially influencing the overall effects of cannabis by offering potential therapeutic benefits [24,25]. Caryophyllene acts as an agonist at CB_2_ receptors, PPAR-α, and PPAR-γ, and inhibits mitogen-activated protein kinase (MAPK), contributing to analgesic, anti-inflammatory, and neuroprotective effects, as well as attenuation of chemotherapy-induced peripheral neuropathy [26,27]. Limonene functions as an agonist at 5-HT1A and TRPA1 receptors while inhibiting nuclear factor-κB (NFκB) and acting as an agonist at A2A receptors, demonstrating antistress, anxiolytic, antidepressant, analgesic, and anti-inflammatory properties [4,28,29]. Pinene inhibits MAPK and NFκB, exerting anti-inflammatory anticancer activity [4,30,31]. Myrcene acts as an agonist at TRPV1 and A_2A_ receptors, providing analgesic effects [4,32], while linalool acts as an agonist at A_1A_, A_2A_, and GABA-A receptors, demonstrating analgesic and anxiolytic effects and anticancer properties [4,29,33]. Flavonoids found in cannabis encompass diverse classes such as flavones (e.g., apigenin and luteolin), flavonols (e.g., quercetin and kaempferol), flavanols (e.g., catechin), and, to a lesser extent, flavanones and anthocyanins. Flavonoids in cannabis interact indirectly with the endocannabinoid system, potentially enhancing endocannabinoid signaling by inhibiting enzymes like FAAH [34]. These compounds also exhibit anti-inflammatory and antioxidant properties [35,36]. Additionally, flavonoids may modulate pain perception and inflammation through interactions with TRP channels [37]. The cannabis plant also produces cannabis-specific flavonoids, such as cannflavin A and cannflavin B, which have been shown to inhibit prostaglandin release, indicating their anti-inflammatory effects [38]. Other plants may also produce similar or identical secondary metabolites to those found in the cannabis plant. Cannabinoids with cannabinoid backbones, such as grifolic acid and daurichromenic acid, are synthesized by various Rhododendron species, including *Rhododendron dauricum* L., *Rhododendron adamsii* Rehder, and Rhododendron anthopogonoides Maxim [39,40,41]. Compounds known as amorfrutins, featuring a cannabinoid backbone with an aralkyl side chain, are present in *Helichrysum umbraculigerum* Less., Glycyrrhiza foetida Desf., and *Amorpha fruticosa* L. [42,43]. Moreover, heterologous biosynthesis methods offer a promising avenue for the production of cannabinoids in Nicotiana benthamiana or Saccharomyces cerevisiae [44]. By introducing genes responsible for cannabinoid biosynthesis into these organisms, they can potentially synthesize cannabinoids such as Δ^9^-tetrahydrocannabinol and cannabidiol, presenting a cost-effective and efficient approach for cannabinoid production in industrial settings. The presence of essential oils and flavonoids is also not limited to cannabis. These compounds are ubiquitously found in diverse plant species across various botanical families. Plant families such as *Lamiaceae*, *Rutaceae*, *Myrtaceae*, *Asteraceae*, and *Lauraceae* are recognized for their abundant reservoirs of essential oils [45]. Similarly, families including *Fabaceae*, *Rosaceae*, *Rutaceae*, and *Asteraceae* are known for their richness in flavonoids [46].

Ongoing research is exploring the antimicrobial potential of Cannabis sativa. Cannabis may offer a promising revolution for addressing antibiotic resistance in bacteria, which is a growing concern in the field of medicine. Cannabinoids may exhibit antibacterial effects against both Gram-positive and Gram-negative strains. Luz-Veiga et al. studied the antimicrobial potential of CBD and CBG, revealing minimal inhibitory concentrations (MIC) against various pathogens [47]. For CBD, the results were as follows: *Staphylococcus aureus* (10 µM), *Staphylococcus epidermidis* (10 µM), *Streptococcus pyogenes* (25 µM), *Propionibacterium acnes* (500 µM), *Listeria innocua* (25 µM), *Pseudomonas aeruginosa* (750 µM), *Escherichia coli* (750 µM), and *Candida albicans* (200 µM). The MIC for CBG were as follows: *Staphylococcus aureus* (25 µM), *Staphylococcus epidermidis* (25 µM), *Streptococcus pyogenes* (50 µM), *Propionibacterium acnes* (10 µM), *Listeria innocua* (50 µM), *Pseudomonas aeruginosa* (400 µM), *Escherichia coli* (500 µM), and *Candida albicans* (400 µM). sCO_2_ extracts from industrial hemp inflorescences showed inhibitory activities against *Escherichia coli*, *Pseudomonas aeruginosa*, *Bacillus subtilis*, and *Staphylococcus aureus* in concentrations from 10.42 µg/mL to 66.03 µg/mL [48]. The sCO_2_ extract with the highest content of CBD and that was rich in α-pinene, β-pinene, β-myrcene, and limonene was the most effective. In another study, methanol extract from the whole cannabis plant exhibited antibacterial effects, with MIC of 12.5 µg/mL for *Pseudomonas aeruginosa*, 25 µg/mL for *Escherichia coli*, and 50 µg/mL for *Staphylococcus aureus* [49]. Cannabis constituents may exhibit antimicrobial effects against a strain of bacteria that has developed resistance to many commonly used antibiotics, including a methicillin-resistant variant of *Staphylococcus aureus*, i.e., MRSA (MIC of hydro-alcoholic extract of *Cannabis sativa*—50 µg/mL) [50]. Ethanolic extracts from *Cannabis sativa* L. obtained from *Cannabis sativa* L. plants, collected during the growth cycle spanning the 5th to the 13th weeks, showed minimum inhibitory concentrations against *Staphylococcus aureus*, including antibiotic-resistant strains ranging from 32 to 64 µg/mL [51]. *Cannabis sativa* essential oil can eradicate bacterial biofilm as the minimum biofilm eradication concentration against Staphylococcus aureus (from a wound, vaginal swab of a pregnant woman, pharyngeal swab of a male patient, and urinary specimen of a male patient) ranging from 16 to 24 mg/mL [52].

A crucial aspect in combating bacterial infections is the strength of the immune system. A robust immune system is capable of recognizing bacterial invaders effectively. It can distinguish between self and non-self, identifying pathogens like bacteria and initiating a response to combat them [53]. Immune cells, such as macrophages and neutrophils, play a critical role in engulfing and digesting bacteria through a process called phagocytosis. A strong immune system ensures these cells are active and efficient in clearing bacterial threats. B cells, a type of white blood cell, produce antibodies that specifically target and neutralize bacteria [54]. The immune system can generate an adequate antibody response to combat bacterial infections as it releases cytokines in response to infection [55]. After an initial encounter with a specific bacterial pathogen, the immune system can develop memory cells (memory B cells and memory T cells), which allow the immune system to mount a quicker and more effective defense if the same bacteria are encountered again in the future [56]. The immunomodulatory properties of cannabis are primarily attributed to its interaction with cannabinoid receptors and have received attention in scientific research. Cannabinoids exert anti-inflammatory and immunomodulatory effects by modulating cellular proliferation and cytokine production [57,58]. CBD shows anti-inflammatory effects by activating CB1 and CB2 receptors located in both the central nervous system and immune cells while also interacting with TRPV1, A2A, and PPAR-γ receptors to further modulate immune response [59,60]. CBD demonstrates immunosuppressive properties by directly suppressing immune cell activation, inducing apoptosis, and promoting regulatory cells, which subsequently exert control over other immune cell targets [60,61]. THC exhibits a broad range of immunomodulatory activities, impacting T cell function, cell proliferation, accessory cell function, and cytokine production [62,63,64].

Cannabis secondary metabolites are poorly soluble in water and have poor bioavailability, which limits their pharmacological potential [65,66]. Developing delivery systems is essential for enhancing their therapeutic potential. So far, various delivery systems, including nanostructured lipid carriers for encapsulating cannabinoids (URB597, AM251, and rimonabant), zeolite–thymol composites, microencapsulated cannabidiol in liposomes, and poly(lactic-co-glycolic acid) microparticles loaded with cannabidiol were prepared [67,68,69,70]. These systems have impacted stability and antimicrobial potential and offer implementation in food and personal care applications, such as sanitary materials or biomedical applications. Thus, current research studied the antimicrobial activity of *Cannabis sativa* systems known to increase solubility and apparent permeability after oral administration, aiming to impact the bioavailability and efficacy of cannabis constituents as it was shown in our previous article [71]. *Cannabis sativa* extract, Henola variety, has demonstrated neuroprotective activity in our earlier studies [71,72]. The gut–brain axis, along with interactions with gut microbiota, significantly influences neuroprotective effects [73]. Gut microbiota may impact brain health through neurotransmitter production, immune modulation, regulation of inflammatory states, and influence on gut permeability [74]. It might be crucial for neuroprotection and reducing the risk of various neurodegenerative diseases [75]. Bearing in mind the link between neuroprotective, antioxidant, anti-inflammatory effects and the link to the microbiome, this paper aimed to investigate the antimicrobial and probiotic properties of *Cannabis sativa* inflorescences extract Henola variety (HiE) and its delivery systems with polyvinyl caprolactam–polyvinyl acetate–polyethylene glycol graft copolymer (Soluplus, Sol), magnesium aluminometasilicate (Neusilin US2, Neu), and hydroxypropyl-β-cyclodextrin (HP-β-CD), and their immunomodulatory and immunostimulatory effects.

## 2. Results

### 2.1. High-Performance Liquid Chromatography Analysis of the Extract and Systems

The extract was prepared by the supercritical CO_2_ extraction from Henola inflorescences, achieving an extraction yield of ~16.74%. Following this, cannabinoid delivery systems were prepared with Sol, Neu, and HP-β-CD as model carriers by the solvent-evaporation method. Ultra-high-performance liquid chromatography with diode array detector (HPLC-DAD) analysis was conducted on both the extract and the systems to quantify the cannabinoid content. The results are shown in Table 1. In the extract, CBD was at the level of 6.04 ± 0.08 mg/g plant material, CBDA was at 2.03 ± 0.07 mg/g plant material, while CBC was at 0.24 ± 0.01 mg/g plant material. The HiE-HP-β-CD system exhibited the highest content of all three cannabinoids: cannabidiol (CBD), cannabidiolic acid (CBDA), and cannabichromene (CBC). The highest content of cannabinoids in this system could potentially lead to improved bioavailability and therapeutic efficacy, thus showing its superiority over other systems.

### 2.2. Antimicrobial and Probiotic Properties

HiE and its systems underwent activity tests against 20 microorganisms comprising both pathogenic and probiotic strains. The results showing the antimicrobial and probiotic properties of the systems are presented in Table 2 and Table 3. HiE decreased the number of *Clostridium difficile* ATCC 9689 from 2.5 × 10^2^ CFU mL^−1^ to <10 CFU mL^−1^, while *Listeria monocytogenes* ATCC 7644 decreased from 3.0 × 10^2^ CFU mL^−1^ to <10 CFU mL^−1^. *Enterococcus faecalis* ATTC 29212 colony was increased from 1.1 × 10^2^ CFU mL^−1^ to 3.3 × 10^3^ CFU mL^−1^, whereas *Staphylococcus aureus* ATCC 25,923 colony was reduced from 7.2 × 10^2^ CFU mL^−1^ to <10 CFU mL^−1^ and *Staphylococcus pyogenes* ATCC 19,615 2.9 × 10^2^ CFU mL^−1^ to 1.8 × 10^2^ CFU mL^−1^. HiE decreased the number of *Escherichia coli* ATCC 25,922 from 7.4 × 10^2^ CFU mL^−1^ to <10 CFU mL^−1^, while *Klebsiella pneumoniae* ATCC 31,488 from 9.5 × 10^2^ CFU mL^−1^ to <10 CFU mL^−1^ and *Salmonella typhimurium* ATCC 14,028 from 1.3 × 10^2^ CFU mL^−1^ to <10 CFU mL^−1^. The number of *Pseudomonas aeruginosa* ATCC 27,853 microorganisms was decreased from 8.2 × 10^2^ CFU mL^−1^ to <10 CFU mL^−1^, while *Candida albicans* ATTC 10,231 from 8.4 × 10^2^ CFU mL^−1^ to <10 CFU mL^−1^.

HiE increased the number of *Lactobacillus casei* (ATCC 393) from 3.9 × 10^2^ CFU mL^−1^ to 3.3 × 10^3^ CFU mL^−1^. The number of bacteria in the colony of *Lactobacillus brevis* (ATCC 8287) was increased from 5.3 × 10^2^ CFU mL^−1^ to 5.5 × 10^4^ CFU mL^−1^. The HiE-HP-β-CD system increased the number of microorganisms from all studied strains (from 100 to 100,000 times). HiE Neu increased the number of *Lactobacillus rhamnosus* about 10 times. HiE-Sol increased the number of *Lactobacillus rhamnosus*, *Pediococcus pentosaceus*, *Lactococcus lactis*, *Lactobacillus fermentum*, and *Streptococcus thermophilus* about 10 times.

Extract from cannabis exhibits antimicrobial properties against both potentially pathogenic microorganisms and probiotic microorganisms, which are beneficial for health. The addition of HP-β-CD stimulates the growth of both pathogenic and probiotic microorganisms. Cyclodextrin serves as a carbon source for microorganisms; hence, this observation is understandable. The other two carriers did not exhibit prebiotic potential. However, statistically significant differences are only observed at a concentration of 100 μg/mL in the system.

### 2.3. Evaluation of Immunomodulatory and Immunostimulatory Properties

**Figure 1:**
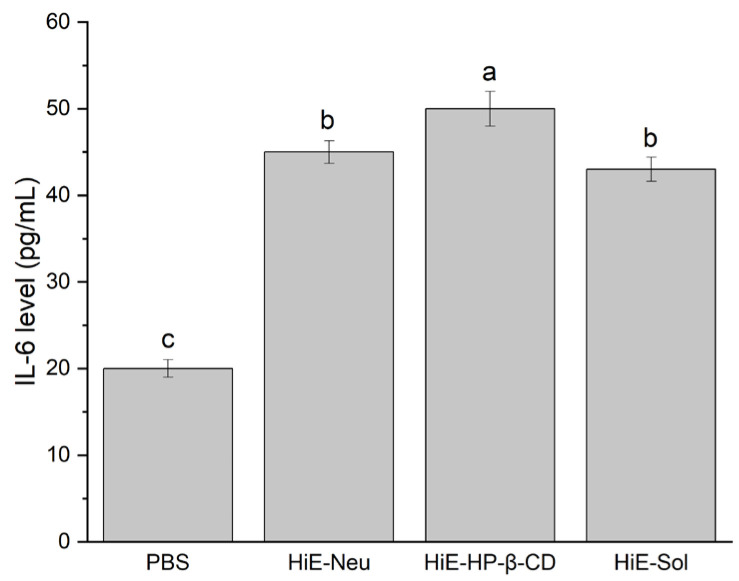
IL-6 levels after 18-hour stimulation of Caco-2 cells with PBS, HiE-Neu, HiE-HP-β-CD, and HiE-Sol. Different letters (a–c) within the bars indicate statistical difference (*p* < 0.05).

**Figure 2:**
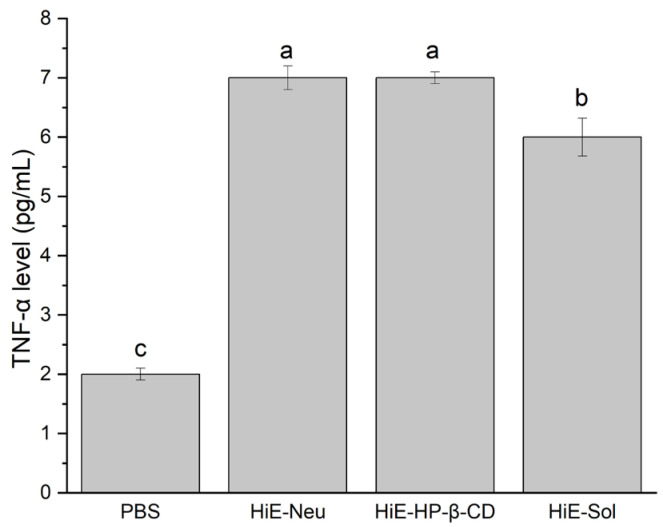
TNF-α level after 18 h stimulation of Caco-2 cells with systems, where 1. PBS, 2. HiE-Neu, 3. HiE-HP-β-CD, 4. HiE-Sol. Different letters (a–c) within the bars indicate statistical difference (*p* < 0.05).

**Figure 3:**
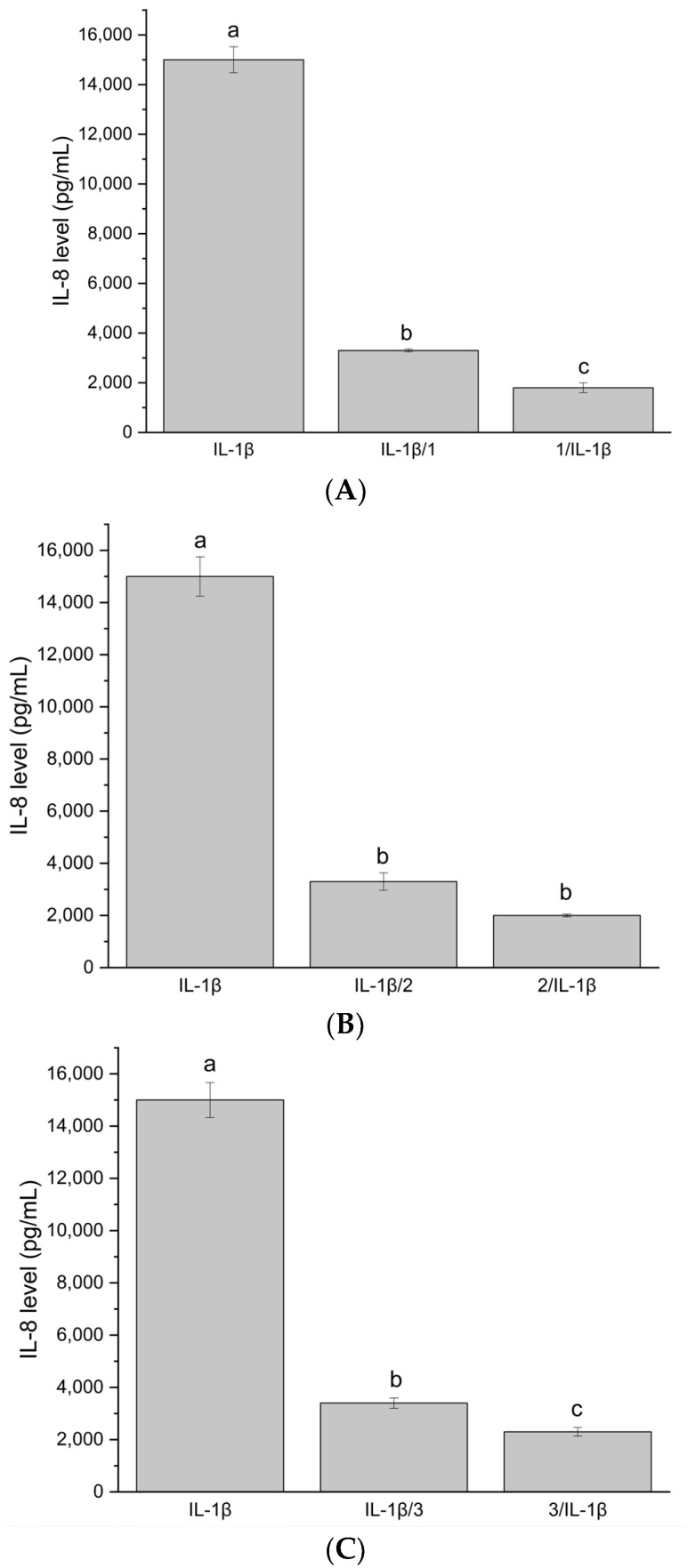
(**A**) IL-8 level after stimulation of Caco-2 cells with system HiE-Neu at a concentration of 100 μg/mL. (**B**) IL-8 level after stimulation of Caco-2 cells with system HiE-HP-β-CD at a concentration of 100 μg/mL. (**C**) IL-8 level after stimulation of Caco-2 cells with system HiE-Sol at a concentration of 100 μg/mL. Different letters (a–c) within the bars indicate statistical difference (*p* < 0.05).

Due to the antimicrobial properties of the tested systems, they were subjected to verification regarding their immunomodulatory and immunostimulatory properties (Figure 1, Figure 2 and Figure 3). The research hypothesis posited that the systems at a concentration of 100 μg/mL possess properties conducive to stimulating the immune system. The induction of interleukin (IL)-6 and tumor necrosis factor (TNF-α) cytokines under the influence of systems of hemp extract was determined. In vitro induction of inflammation caused by proinflammatory cytokines was conducted to ascertain whether the system exhibits protective properties (administration of the systems before induction of inflammation) as well as potential therapeutic effects (administration of the systems after induction of inflammation). The level of IL-8 was measured.

Induction of IL-6 production was observed. The level of IL-6 following stimulation with the studied systems was higher compared to PBS alone (negative control).

Another indicator was the TNF-α factor, which was produced by Caco-2 cells in the case of systems at a concentration of 100 μg/mL. The obtained response at the level of the measured marker was similar for all three tested systems; therefore, it should be considered that a dose of 100 μg/mL is sufficient to achieve the effect of immunomodulation and immunostimulation.

The next stage of the study involved assessing whether the systems can reduce the level of IL-8 produced as a result of administering proinflammatory IL-1β to Caco-2 cells, encompassing two models: administration of the system before induction of inflammation (prior to administering IL-1β cytokine to Caco-2 cells) and administration of the system after induction of inflammation (administration after treating Caco-2 cells with IL-1β cytokine). To achieve this, the Caco-2 cell line was maintained in culture up to passage 20 (culture and stimulation conditions as above) and, at this passage, an experiment was conducted involving the administration of system samples to Caco-2 cells at a concentration of 100 μg/mL.

The inflammatory state induction model was obtained by administering 10 ng/μL of IL-1β cytokine to Caco-2 cells. The effectiveness of inducing inflammation was measured by the level of proinflammatory IL-8 produced by Caco-2 cells (ELISA test, B&D). The experiment was conducted in two models:

Stimulation of Caco-2 cells with investigated system samples for 18 h, followed by IL-1β administration for another 18 h.

Stimulation of Caco-2 cells with IL-1β for 18 h, followed by administration of investigated system samples for another 18 h.

In vitro induction of inflammation via proinflammatory cytokines was conducted to assess the protective properties of the systems when administered before inflammation induction, as well as its potential therapeutic effects when administered after inflammation induction.

Additionally, Caco-2 cells were stimulated with only the investigated system samples to verify whether the samples induce IL-8 production. The experiment was repeated three times (three biological replicates), with each sample applied in two technical replicates.

The systems at 100 μg/mL did not induce IL-8 production. The test functions correctly because, after administering only IL-1β, the IL-8 level is high, approximately 15,000 pg/mL. The tested samples at a concentration of 100 μg/mL exhibit both protective and therapeutic effects in the in vitro model. The IL-8 level is significantly reduced compared to the IL-8 level after administering only IL-1β. The protective effect is stronger. The investigated systems demonstrate immunomodulatory and immunostimulatory effects at a concentration of 100 μg/mL. No significant increase in IL8 synthesis was observed, suggesting that the existing inflammation was not stimulated and that the applied systems did not induce, e.g., allergic reactions. It is important to note that the differences between the studied systems were not statistically significant.

## 3. Discussion

Microbiological studies have shown that HiE-Neu and HiE-Sol systems inhibit the growth of potentially pathogenic microorganisms. In contrast, HiE-HP-β-CD showed growth of potentially pathogenic microorganisms as well as those with probiotic potential due to the fact that cyclodextrin contained in the system provides a carbon source for microorganisms and stimulates their proliferation. In the case of probiotic microorganisms, no stimulation of their growth was observed in the case of the HiE-Neu system. In this case, the growth of microorganisms was completely inhibited. Given the chemical composition of the systems studied and the literature studies, it can be assumed that the antimicrobial activity of the systems studied is primarily due to the presence of cannabinoids, the properties of which were reported by the literature in the 1950s [76,77]. In the past, the bactericidal properties of hemp were studied without knowledge of its phytochemical composition [78]. Later work indicates that essential oils extracted from five different varieties of *C. sativa* were evaluated for their antimicrobial properties against Gram-negatives and Gram-positives. Compounds that contribute to the antimicrobial nature of *C. sativa* were also identified. These include trans-β-cymene, myrcene, and trans-caryophyllene [79]. The method of preparation of the plant material is also not without significance. Compared to aqueous extracts, acetone extracts showed better bactericidal properties and the most sensitive species were bacteria of the species *V. cholera* and *P. aeruginosa* [50]. Experimental results showed that hemp extracts significantly inhibited the growth of *S. aureus* 25923. Other findings support the claim that the antimicrobial properties of *C. sativa* plants are low against Gram-positive bacteria [80]. Other authors have studied the effect of hemp seed oil-based emulsions. For example, the activity of the oil-based emulsion against *E. coli* was virtually nonexistent, which could be due to a higher concentration of α-linolenic acid or, more likely, the removal of Δ^9^-THC during the refining process [81]. The *C. sativa* extract was tested against various types of antibiotic-resistant bacteria, such as MRSA, using a disk diffusion method. A zone of inhibition of clinical isolates ranging from 9 to 15 mm (vancomycin diameter 13–24 mm) was observed. However, it is crucial to know the antimicrobial mechanism of cannabinoids and it is probably related to cell membrane permeability. Thus, in the case of *L. monocytogenes*, the integrity of wall structures was disrupted by the terpene limonene, which caused cell lysis [82]. Similar changes to those induced by β-caryophyllene were observed in the membrane of the bacterium *Bacillus cereus* [83]. It should be pointed out that the inner membrane of Gram-negative bacteria is permeable, which allows CBG to act in a manner comparable to that of Gram- positive bacteria [84]. Microscopic evaluation of the efficacy of CBCA on the growth of B. subtilis showed that it induces a change in the bacterial membrane and nucleoid [85]. In vitro studies have shown that CBD causes depolarization of the membrane of *S. aureus* and disrupts the membrane potential of the bacteria [84]. The combination of CBD and bacitracin can cause various defects in cell division and cell envelope abnormalities. The abnormalities were thought to be caused by the loss of genes that regulate cell division [86]. Another mode of action of cannabinoids, which can be used to alter cell communication, is to block the release of membrane vesicles by bacteria. Although it has been shown that CBD can block the release of membrane vesicles from the pathogen, this effect was not significant in the presence of *S. aureus* [87]. In one study, a radio-labeled synthesis assay in *S. aureus* RN42200 revealed different pathways leading to protein, DNA, and RNA synthesis [88]. This suggests that the rapid bactericidal action is aimed at shutting down these pathways [89]. Decreased lipid synthesis was observed at concentrations below MIC, supporting the hypothesis of membrane mechanisms [90]. The experimental results suggest that various cannabinoid-containing systems have diverse effects on the gut microbiota. For example, HiE exhibits potent antibacterial activity, inhibiting the growth of pathogenic microorganisms, while the HiE-HP-β-CD system shows probiotic effects, promoting the proliferation of beneficial microorganisms. Therefore, depending on the state of the microbiota, *C. sativa* may be beneficial in restoring the gut balance necessary for the proper functioning of the gut–brain axis.

The cellular response upon exposure to Henola *Cannabis sativae* extract, both independently and in combination with various carriers (polyvinyl caprolactam–polyvinyl acetate–polyethylene glycol graft copolymer, magnesium aluminometasilicate, and hydroxypropyl-β-cyclodextrin) was analyzed. The studied extract and systems elicit a response, stimulating the immune system to activate. Consequently, in scenarios of ongoing inflammation, such as in a wound, the introduction of the products mentioned above is likely to prompt further defensive reactions within the body. Admittedly, our evaluation is somewhat simplified and limited, focusing solely on two components of the cellular response, namely IL-6 and TNF. However, these markers are indicative of most inflammatory conditions and can be considered as consistently present in any inflammatory process. Additionally, our study used a well-established and extensively characterized cell line, commonly used in such investigations. This choice was necessitated by the significant cost associated with broader studies, prompting us to prioritize the most representative indicators.

The second aspect examined involved the induction of inflammation through the administration of proinflammatory IL-1β to the cells, a naturally occurring agent known to incite inflammatory responses in biological systems. Our systems were applied to this pre-existing inflammatory condition. The marker used to measure cellular responsiveness was IL-8, a cytokine renowned for its role in stimulating the migration of immune cells within the body, including T lymphocytes, neutrophils, and monocytes, thereby functioning in a defensive capacity. Furthermore, IL-8 prompts the release of histamine by basophils, thereby potentially eliciting allergic reactions. Our results indicate that there was no discernible increase in IL-8 synthesis, suggesting that the systems did not further stimulate the existing inflammation, and no allergic reactions were induced. Moreover, it is worth highlighting that CBD, a non-psychoactive metabolite derived from *Cannabis sativa* L., is renowned for its potent anti-inflammatory properties. CBD has been shown to modulate the immune response in both human cell cultures and animal models, acting as an exogenous ligand for multiple receptors, including CB_1_, CB_2_, 5-HT1A, PPAR-γ, A_2A_, and GPR55, which are expressed by various cell types [60,91,92]. Signals from these multiple cannabinoid/receptor interactions reduce the secretion of proinflammatory cytokines (IL-1β, IL-8, IL-10, IL-12, TNF-α, and interferon-gamma (IFN-γ)) and balance the immune response.

It is important to highlight that the research was primarily intended to explore the potential of the designed systems in stimulating and modulating the immune system. The aim was to investigate whether a cause-and-effect relationship exists between these systems and immune responses, a connection that was indeed established. While further validation of these preliminary findings is essential, the results obtained suggest a promising direction for future research. It is noteworthy that the existing literature on the subject elucidates various mechanisms of action associated with CBD. A substantial body of evidence underscores CBD’s anti-inflammatory properties and its role in enhancing immunomodulation and immunostimulation. However, it is worth noting that the efficacy of these effects is contingent upon the concentration/dosage of CBD and factors inherent to the cell culture methodology. Notably, findings from other researchers corroborate the results obtained in this study that CBD stimulates the production of cytokines [93,94,95]. Reports also reference the so-called differential mechanism, contingent upon the intensity of immune stimulation. An elucidation of this mechanism can be found in the literature, likely associated with fluctuations in intracellular calcium concentrations and correlation with the expression of the nuclear factor of activated T cells transcription factor [94]. It can be assumed that intracellular calcium concentration plays an important role in the modulation of the immune system [96,97].

## 4. Materials and Methods

### 4.1. Materials

*Cannabis sativa* plant material, the Henola variety, was generously provided by the Experimental Station for Cultivar Testing in Chrząstowo, a division of the Research Centre for Cultivar Testing in Słupia Wielka. The plant samples were harvested after hemp plants reached the maturation phase, i.e., from the moment of seed formation to the first seed. Following collection, we separated two 500 g samples, subjecting them to a drying process until they reached an entirely dry mass. This drying procedure spanned 20 hours in total, with the oven temperature initially not exceeding 50 °C for the initial six hours and subsequently maintained at 105 °C for the remaining 14 h of the drying process.

Food-grade CO_2_ was purchased from Air Liquide Polska (Cracow, Poland), while Soluplus^®^ (a polyvinyl caprolactam–polyvinyl acetate–polyethylene glycol graft copolymer) was generously provided by BASF SE (Ludwigshafen, Germany). Neusilin US2 (magnesium aluminometasilicate) was kindly supplied by Fuji Chemical Industry (Minato, Tokyo). Hydroxypropyl-β-cyclodextrin (molar substitution 0.8, Mw~1.460) was purchased from Sigma-Aldrich (Poznan, Poland). Cannabinoid standards, including CBD, CBDA, and CBC, were procured from Sigma-Aldrich in Poznan, Poland. Trifluoroacetic acid and acetonitrile (HPLC grade) were purchased from Merck (Darmstadt, Germany). Caco-2 cell line was obtained from American Tissue Culture Collection (USA).

The microbiological research material consisted of strains of microorganisms originating from international collections of pure cultures (Table S1, Supplementary Materials). The microorganisms were stored in a frozen state at a temperature of −20 °C.

### 4.2. Preparation of the Systems

The extraction process and preparation of the systems were performed as presented in our previous paper [71,72]. The extract from *Cannabis sativa* inflorescences was obtained by a dynamic supercritical CO_2_ extraction process using the SFT-120 apparatus (shim-pol, Izabelin, Polska). Specifically, 6.5 g of dried plant material was placed into the extraction vessel and extracted under conditions of 6000 psi at 50 °C with 250 mL of CO_2_. The extraction yield was calculated as the mass of extract obtained and subjected to drying (to remove any water from the eventually frozen needle) (g) divided by the mass (g) of plant material placed in the extractor and expressed as a percentage (%). Subsequently, the extract underwent vacuum drying and weighing, and then it was suspended in methanol, winterized, and filtered. For the preparation of fluid extract (HiE), carriers (Neu, Sol, and HP-β-CD) were incorporated at a 1:1 mass ratio relative to the initial weight of the extract. The choice of carriers was based on our previous article and was enriched in hydroxypropyl-β-cyclodextrin as it might be a source of carbon for probiotic bacteria [98]. Moreover, hydroxypropyl-β-cyclodextrin was the greatest cyclodextrin to improve cannabinoids’ solubility in preliminary studies. The resulting systems were dried using a rota-vapor (BUCHI System, Flawil, Switzerland) at 50 °C until they reached a dry state and were then ground using a mortar and pestle.

### 4.3. Chromatographic Analysis

The cannabinoid profile (CBD, CBDA, and CBC) of the extract and the systems was analyzed using ultra-high-performance liquid chromatography with the diode array detector (HPLC-DAD)-validated method, Shimadzu Corp. (Kyoto, Japan). The previously described and validated method by the authors was used [35,71,72]. The mobile phase consisted of 0.1% trifluoroacetic acid and acetonitrile (41:59, *v*/*v*), and the column CORTECS Shield RP18 (2.7 µm; 150 mm × 4.6 mm, Cortecs, St. Paul, MN, USA) was used. The flow rate was 2.0 mL/min and the column temperature was maintained at 35 °C. The injection volume was 10.0 µL and detection was set at 228 nm, with a retention time of approximately 5.8 min for CBD, 6.4 min for CBDA, and 14.6 min for CBC. The analysis time was 50 min. The LabSolutions LC software from Shimadzu Corp. (version 1.86 SP2, Kyoto, Japan) was used to obtain chromatograms.

### 4.4. Evaluation of Antimicrobial and Probiotic Properties of the Extract and the Systems

The microbiological test material used in this study comprised microbial strains sourced from globally recognized repositories of pure cultures. These microorganisms were maintained in a frozen state at −40 °C, suspended in a medium containing 20% glycerol to ensure viability and preservation of genetic integrity. The selection of microbial species for this investigation adhered to rigorous criteria aligned with scientific standards and the objectives of the research: *Clostridium difficile* ATCC 9689, *Listeria monocytogenes* ATCC 7644, *Enterococcus faecalis* ATTC 29212, *Staphylococcus aureus* ATCC 25923, *Staphylococcus pyogenes* ATCC 19615, *Escherichia coli* ATCC 25922, *Klebsiella pneumoniae* ATCC 31488, *Salmonella typhimurium* ATCC 14028, *Pseudomonas aeruginosa* ATCC 27853, *Candida albicans* ATTC 10231, *Lactobacillus acidophilus* 4356, *Lactobacillus casei* ATCC 393, *Lactobacillus plantarum* ATCC 14917, *Lactobacillus brevis* ATCC 8287, *Lactobacillus rhamnosus* GG ATCC 53103, *Lactobacillus reuteri* ATCC 5289, *Pediococcus pentosaceus* ATCC 25745, *Lactococcus lactis* ATCC 11955, *Lactobacillus fermentum* LF 2 (LMG 27299), and *Streptococcus thermophilus* FP 4 (DSMZ 18616).

In the initial stage of the research, microbial strains were prepared by suspending 0.1 g of lyophilized bacteria in 10 mL of liquid Muller–Hinton enrichment medium. The samples were then incubated at 30 °C (yeasts) or 37 °C (bacteria) for 18 h to activate and proliferate the biomass. After achieving the appropriate turbidity, the biomass was separated from the medium by centrifugation (14,000 rpm for 10 min). The supernatant was discarded and the pellet was resuspended in 10 mL of 0.9% NaCl solution before being centrifuged again. This washing procedure was repeated three times. Subsequently, the biomass was suspended in 0.9% NaCl solution to achieve a microbial concentration of 1.0 × 10^2^ colony-forming units per milliliter (CFU mL^−1^). Simultaneously, a solution of test samples (dissolved in 5% DMSO) was prepared at a concentration of 100 μg/mL. The prepared solution was then inoculated with the prepared microbial suspension. The samples were mixed and incubated at 30 °C (yeasts) or 37 °C (bacteria) for 18 h under appropriate gas conditions for the respective microbial groups. The microbial count was determined both before and after incubation using culture media specific to the microbial group under investigation.

### 4.5. Evaluation of Immunomodulatory and Immunostimulatory Properties of the Systems

Microbial reference strains were purchased from the microbial collection in lyophilized form. In the initial phase of the study, microbial strains were prepared by suspending 0.1 g of lyophilized bacteria in 10 mL of liquid Mueller–Hinton enrichment medium. It is a medium dedicated to microorganisms with special nutritional requirements and is most commonly used in microbiological studies. The samples were then incubated at 30 °C (yeasts) or 37 °C (bacteria) for 18 h to activate and propagate the biomass. After incubation, the biomass was separated from the medium by centrifugation (14,000 rpm for 10 min). The supernatant was discarded and the pellet was resuspended in 10 mL of 0.9% NaCl solution before undergoing centrifugation once more. This procedure was repeated three times. Subsequently, the biomass was diluted in 0.9% NaCl solution to achieve a microbial concentration of 1.0 × 10^2^ colony-forming units per milliliter (CFU mL^−1^)—optical density at 605 nm was measured. A standard curve for determining the relationship between the optical density (absorbance value) and the number of cells expressed in CFU mL^−1^ was prepared before. Concurrently, three solutions of test samples (dissolved in 5% DMSO) were prepared at concentrations of 10, 50, and 100 μg/mL. The prepared dilutions were then inoculated with the prepared microbial suspension. The samples were mixed and incubated at 30 °C (yeasts) or 37 °C (bacteria) for 18 h under appropriate gas conditions suitable for the respective microbial groups. The microbial count was analyzed before and after incubation using media specific to the microbial group under investigation.

### 4.6. Statistical Analysis

Statistical analysis was performed using Statistica 13.3 software (StatSoft Poland, Krakow, Poland). Experimental data were analyzed using the skewness and kurtosis tests to determine the normality of each distribution, and Levene’s test assessed the equality of variances. Statistical significance was determined using a one-way analysis of variance (ANOVA), followed by the Bonferroni post hoc test. Differences were considered significant at *p* < 0.05.

## 5. Conclusions

This study investigated the antimicrobial potential of *Cannabis sativa* extract, Henola variety, and systems with carriers. The extract showed antimicrobial activity against pathogenic microorganisms, suggesting its possible application as support in combating infections. Additionally, the system with hydroxypropyl-β-cyclodextrin may possess prebiotic properties, stimulating the growth of probiotic microorganisms. Furthermore, the investigated systems exhibit immunomodulatory and immunostimulatory effects, with potential therapeutic implications for modulating inflammatory responses. Overall, these findings underscore the multifaceted therapeutic potential of *Cannabis sativa* extracts. The delivery systems might be used as powder-based food additives, but they might also be subjected to formulation studies for the development of an oral dietary supplement.

## Figures and Tables

**Table 1:** The content of cannabinoids: cannabidiol (CBD), cannabidiolic acid (CBDA), and cannabichromene (CBC) in the extract (mg cannabinoid/g of plant material) and the HiE-Neu, HiE-Sol, and HiE-HP-β-CD systems (mg cannabinoid/g of the system).

Cannabinoid	Extract (mg/g)	HiE-Neu System (mg/g)	HiE-Sol System (mg/g)	HiE-HP-β-CD System (mg/g)
Cannabidiol (CBD)	6.04 ± 0.08	8.73 ± 0.08	10.77 ± 0.06	13.87 ± 0.10
Cannabidiolic Acid (CBDA)	2.03 ± 0.07	2.85 ± 0.02	3.60 ± 0.02	4.85 ± 0.03
Cannabichromene (CBC)	0.24 ± 0.01	0.379 ± 0.004	0.323 ± 0.004	0.46 ± 0.01

**Table 2:** Change in the number of potentially pathogenic microorganisms in the medium with a concentration of the system of 100 μg/mL.

System	Strains of Potentially Pathogenic Microorganisms									
	1	2	3	4	5	6	7	8	9	10
	Microorganism Count (CFU mL^−1^)									
HiE-Neu	6.9 × 10^2^ →<10	4.8 × 10^2^ →<10	2.0 × 10^2^ →<10	3.3 × 10^2^ →<10	1.5 × 10^2^ →1.5 × 10^2^	9.0 × 10^2^ →<10	2.1 × 10^2^ →<10	3.6 × 10^2^ →3.1 × 10^2^	7.8 × 10^2^ →2.92 × 10^2^	3.7 × 10^2^ →<10
HiE-HP-β-CD	2.9 × 10^2^ →3.0 × 10^7^	7.7 × 10^2^ →8.2 × 10^6^	2.2 × 10^2^ →3.5 × 10^7^	2.2 × 10^2^ →3.5 × 10^7^	2.2 × 10^2^ →3.5 × 10^7^	3.4 × 10^2^ →5.1 × 10^7^	2.9 × 10^2^ →3.0 × 10^7^	2.0 × 10^2^ →3.3 × 10^6^	2.5 × 10^2^ →3.2 × 10^6^	2.1 × 10^2^ →3.2 × 10^7^
HiE-Sol	2.5 × 10^2^ →<10	1.8 × 10^2^ →<10	2.5 × 10^2^ →3.3 × 10^3^	2.7 × 10^2^ →<10	3.5 × 10^2^ →<10	2.1 × 10^2^ →<10	1.9 × 10^2^ →<10	2.6 × 10^2^ →<10	3.4 × 10^2^ →<10	2.9 × 10^2^ →1.5 × 10^2^

Legend: 1—Clostridium difficile ATCC 9689, 2—Listeria monocytogenes ATCC 7644, 3—Enterococcus faecalis ATTC 29212, 4—Staphylococcus aureus ATCC 25923, 5—Staphylococcus pyrogenes ATCC 19615, 6—Escherichia coli ATCC 25922, 7—Klebsiella pneumoniae ATCC 31488, 8—Salmonella typhimurium ATCC 14028, 9—Pseudomonas aereuginosa ATCC 27853, 10—Candida albicans ATTC 10231.

**Table 3:** Change in the number of probiotic microorganisms in the medium with a concentration of the system of 100 μg/mL.

System	Strains of Microorganisms Potentially Probiotic									
	11	12	13	14	15	16	17	18	19	20
	Microorganism Count (CFU mL^−1^)									
HiE-Neu	2.8 × 10^2^ → <10	2.2 × 10^2^ → <10	2.1 × 10^2^ → <10	3.6 × 10^2^ → <10	2.9 × 10^2^ →6.8 × 10^3^	1.8 × 10^2^ → <10	8.1 × 10^2^ →<10	3.0 × 10^2^ →<10	7.2 × 10^2^ →<10	6.4 × 10^2^ →<10
HiE-HP-β-CD	7.7 × 10^2^ →8.5 × 10^4^	2.2 × 10^2^ →3.5 × 10^7^	3.4 × 10^2^ →5.1 × 10^7^	2.9 × 10^2^ →3.0 × 10^6^	3.4 × 10^2^ →3.3 × 10^7^	1.3 × 10^2^ →3.2 × 10^7^	2.0 × 10^2^ →5.4 × 10^6^	3.8 × 10^2^ →4.8 × 10^7^	1.4 × 10^2^ →3.2 × 10^7^	3.4 × 10^2^ →8.2 × 10^7^
HiE-Sol	4.8 × 10^2^ → <10	2.0 × 10^2^ →<10	2.0 × 10^2^ →5.4 × 10^2^	6.9 × 10^2^ →3.3 × 10^2^	2.7 × 10^2^ →5.2 × 10^3^	5.3 × 10^2^ →3.0 × 10^3^	3.6 × 10^2^ →3.0 × 10^3^	8.3 × 10^2^ →3.0 × 10^3^	1.9 × 10^2^ →3.0 × 10^3^	2.7 × 10^2^ →3.0 × 10^3^

Legend: 11—Lactobacillus acidophilus 4356, 12—Lactobacillus casei ATCC 393, 13—Lactobacillus plantarum ATCC 14917, 14—Lactobacillus brevis ATCC 8287, 15—Lactobacillus rhamnosus GG ATCC 53103, 16—Lactobacillus reuteri ATCC 5289, 17—Pediococcus pentosaceus ATCC 25745, 18—Lactococcus lactis ATCC 11955, 19—Lactobacillus fermentum LF 2 (LMG 27299), 20—Streptococcus thermophilus FP 4 (DSM 18616).

## Data Availability

Data are available in a publicly accessible repository.

## Supplementary Materials

The following supporting information can be downloaded at: https://www.mdpi.com/article/10.3390/antibiotics13040369/s1, Table S1: The microorganisms and growth conditions used in studies investigating antimicrobial and probiotic potential.
