# Supplementary material for: Antimicrobial, Probiotic, and Immunomodulatory Potential of Cannabis sativa Extract and Delivery Systems
Source: Antibiotics (Basel). 2024 Apr 17;13(4):369. doi: 10.3390/antibiotics13040369 (PMC11047504; doi:10.3390/antibiotics13040369)
Supplement: Supplementary file 1 [file antibiotics-13-00369-s001.zip › antibiotics-2920864-supplementary.pdf]

Supplementary materials

Antimicrobial, Probiotic, and Immunomodulatory Potential of *Cannabis sativa* Extract and Delivery Systems

Anna Stasiłowicz-Krzemień [1,*], Daria Szymanowska [1,2], Piotr Szulc [2] and Judyta Cielecka-Piontek [1,3,*]

[1] Department of Pharmacognosy and Biomaterials, Faculty of Pharmacy, Poznan University of Medical Sciences, Rokietnicka 3, 60-806 Poznan, Poland; astasilowicz@ump.edu.pl, jpiontek@ump.edu.pl
[2] Department of Biotechnology and Food Microbiology, Poznan University of Life Sciences, 48 Wojska Polskiego Street, 60-627 Poznan, Poland, daria.szymanowska@up.poznan.pl
[3] Department of Agronomy, Poznań University of Life Sciences, Dojazd 11, 60-632 Poznań, Poland; piotr.szulc@up.poznan.pl
[4] Department of Pharmacology and Phytochemistry, Institute of Natural Fibres and Medicinal Plants, Wojska Polskiego 71b, 60-630 Poznan, Poland
* Correspondence: astasilowicz@ump.edu.pl; jpiontek@ump.edu.pl

Table S1. The microorganisms and growth conditions used in studies investigating antimicrobial and probiotic potential.

Microorganism	Strain	Type of Culture Collection	Culture Media	Growth Conditions
Clostridium difficile	ATCC 9689	American Type Culture Collection	Muller-Hinton	Anaerobic, 37°C, 48 h
Listeria monocytogenes	ATCC 7644	American Type Culture Collection	Muller-Hinton	Facultative anaerobic, 37°C, 48 h
Enterococcus faecalis	ATTC 29212	American Type Culture Collection	Muller-Hinton	Facultative anaerobic, 37°C, 48 h
Staphylococcus aureus	ATCC 25923	American Type Culture Collection	Muller-Hinton	Aerobic, 37°C, 48 h
Staphylococcus pyogenes	ATCC 19615	American Type Culture Collection	Muller-Hinton	Aerobic, 37°C, 48 h
Escherichia coli	ATCC 25922	American Type Culture Collection	Muller-Hinton	Facultative anaerobic, 37°C, 48 h
Klebsiella pneumoniae	ATCC 31488	American Type Culture Collection	Muller-Hinton	Facultative anaerobic, 37°C, 48 h
Salmonella typhimurium	ATCC 14028	American Type Culture Collection	Muller-Hinton	Facultative anaerobic, 37°C, 48 h
Pseudomonas aeruginosa	ATCC 27853	American Type Culture Collection	Muller-Hinton	Facultative anaerobic, 37°C, 48 h
Candida albicans	ATTC 10231	American Type Culture Collection	Muller-Hinton	Aerobic, 37°C, 72 h
Lactobacillus acidophilus	4356	American Type Culture Collection	Muller-Hinton	Facultative anaerobic, 37°C, 48 h
Lactobacillus casei	ATCC 393	American Type Culture Collection	Muller-Hinton	Facultative anaerobic, 37°C, 48 h
Lactobacillus plantarum	ATCC 14917	American Type Culture Collection	Muller-Hinton	Facultative anaerobic, 37°C, 48 h
Lactobacillus brevis	ATCC 8287	American Type Culture Collection	Muller-Hinton	Facultative anaerobic, 37°C, 48 h
Lactobacillus rhamnosus GG	ATCC 53103	American Type Culture Collection	Muller-Hinton	Facultative anaerobic, 37°C, 48 h
Lactobacillus reuteri	ATCC 5289	American Type Culture Collection	Muller-Hinton	Facultative anaerobic, 37°C, 48 h
Pediococcus pentosaceus	ATCC 25745	American Type Culture Collection	Muller-Hinton	Aerobic, 37°C, 48 h
Lactococcus lactis	ATCC 11955	American Type Culture Collection	Muller-Hinton	Facultative anaerobic, 37°C, 48 h
Lactobacillus fermentum LF 2	LMG 27299	Belgian Coordinated Collections of Microorganisms	Muller-Hinton	Facultative anaerobic, 37°C, 48 h

Streptococcus thermophilus FP 4	DSMZ 18616	German Collection of Microorganisms and Cell Cultures	Muller-Hinton	Aerobic, 37°C, 48 h
